# Failure of anti tumor-derived endothelial cell immunotherapy depends on augmentation of tumor hypoxia

**DOI:** 10.18632/oncotarget.2015

**Published:** 2014-05-26

**Authors:** Annalisa Pezzolo, Danilo Marimpietri, Lizzia Raffaghello, Claudia Cocco, Angela Pistorio, Claudio Gambini, Michele Cilli, Alberto Horenstein, Fabio Malavasi, Vito Pistoia

**Affiliations:** ^1^ Laboratorio di Oncologia, Istituto Giannina Gaslini, Genova, Italy; ^2^ Unità di Epidemiologia e Biostatistica Istituto Giannina Gaslini, Genova, Italy; ^3^ Laboratorio di Anatomia Patologica Istituto Giannina Gaslini, Genova, Italy; ^4^ Animal Research Facility, IRCCS-AOU San Martino-IST Istituto Nazionale per la Ricerca sul Cancro, Genova, Italy; ^5^ Laboratorio di Immunogenetica, Università di Torino, Italy

**Keywords:** epithelial-mesenchymal transition, hypoxia, neuroblastoma, tumor-derived endothelial cells, vascular mimicry

## Abstract

We have previously demonstrated that Tenascin-C (TNC)^+^ human neuroblastoma (NB) cells transdifferentiate into tumor-derived endothelial cells (TDEC), which have been detected both in primary tumors and in tumors formed by human NB cell lines in immunodeficient mice. TDEC are genetically unstable and may favor tumor progression, suggesting that their elimination could reduce tumor growth and dissemination. So far, TDEC have never been targeted by antibody-mediated immunotherapy in any of the tumor models investigated.

To address this issue, immunodeficient mice carrying orthotopic NB formed by the HTLA-230 human cell line were treated with TDEC-targeting cytotoxic human (h)CD31, that spares host-derived endothelial cells, or isotype-matched mAbs. hCD31 mAb treatment did not affect survival of NB-bearing mice, but increased significantly hypoxia in tumor microenvironment, where apoptotic and proliferating TDEC coexisted, indicating the occurrence of vascular remodeling.

Tumor cells from hCD31 mAb treated mice showed i) up-regulation of epithelial-mesenchymal transition (EMT)-related and vascular mimicry (VM)-related gene expression, ii) expression of endothelial (i.e. CD31 and VE-cadherin) and EMT-associated (i.e. Twist-1, N-cadherin and TNC) immunophenotypic markers, and iii) up-regulation of high mobility group box-1 (HMGB-1) expression. In vitro experiments with two NB cell lines showed that hypoxia was the common driver of all the above phenomena and that human recombinant HMGB-1 amplified EMT and TDEC trans-differentiation.

In conclusion, TDEC targeting with hCD31 mAb increases tumor hypoxia, setting the stage for the occurrence of EMT and of new waves of TDEC trans-differentiation. These adaptive responses to the changes induced by immunotherapy in the tumor microenvironment allow tumor cells to escape from the effects of hCD31 mAb.

## INTRODUCTION

Tumor growth is critically dependent on adequate blood supply provided by newly formed endothelial micro-vessels (EM) [1-3]. Tumor cells themselves may contribute to vascularization by assembling into vascular-like channels according to a process referred to as vascular mimicry (VM). [4].

Neuroblastoma (NB) is an embryonal tumor arising from sympathetic neuronal progenitor cells that accounts for 15% of all pediatric cancer deaths [5-7]. We previously identified in primary NB tumors, as well as in orthotopic tumors formed in immunodeficient mice by different human NB cell lines, tumor-derived endothelial cells (TDEC) harboring *MYCN* amplification as the NB cells from which they originated [8-10]. More recently, we have identified perivascular NB progenitor cells expressing Tenascin C (TNC) on the cell surface, that displayed a high degree of plasticity and served as TDEC progenitors [10]. TDEC are genetically unstable and contribute to chemo-resistance and tumor progression [11].

A hypoxic microenvironment is of pivotal importance for tumor growth. Hypoxia inducible factors regulate hypoxia responsive genes and play critical roles in tumor invasion, metastasis, and chemoresistance [12].

Epithelial-mesenchymal transition (EMT) is an embryonic process leading to the loss of cell-cell contact, repression of E-cadherin expression and increased cell motility. EMT can also occur in cancer cells, in which it is associated with resistance to chemotherapeutic drugs and radiation [13], and increased stemness, motility, invasiveness, as well as angiogenic and metastatic ability [13, 14]. An hypoxic tumor microenvironment is one of the major EMT inducers [15, 16].

We have hypothesized that selective elimination of TDEC might reduce tumor growth. To address this issue, we have here selectively targeted TDEC in an orthotopic mouse model of human NB using a cytotoxic hCD31 mAb that does not react with mouse endothelial cells (mEC). Our findings demonstrate that hCD31 mAb-induced enhancement of tumor hypoxia activates i) EMT and ii) trans-differentiation of malignant cells into TDEC, both of which in turn account for the failure of such therapeutic approach.

## RESULTS

### Tumor-derived endothelial cells (TDEC) contribute to tumor vascularization in an orthotopic mouse model of human NB

Immunodeficient mice were inoculated in the adrenal gland with the human NB cell line HTLA-230 that, according to previous studies from our group [8-10], best mimics human NB growth and progression. Mice were treated with the hCD31 cytotoxic Moon-1 mAb [17] or isotype-matched control mAb. Supplementary Fig. 1 shows that the hCD31 mAb Moon-1 stained human TDEC, but not mEC.

All experiments were performed with tumors harvested eighteen days after NB cell inoculation. Such time point was selected on the ground of our previous studies showing that tumor-derived and mouse-derived EM, although displaying different kinetics of formation, are present in similar proportions after approximately two weeks from HTLA-230 NB cell inoculation [10].

**Figure 1:**
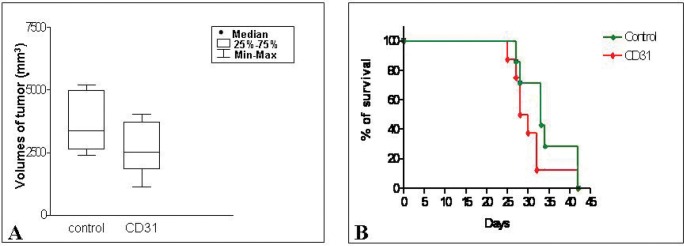
Effect of hCD31 mAb treatment on tumor growth and survival in orthotopic NB bearing mice A) Tumors from mice treated with hCD31 mAb (n = 21) and sacrificed after 18 days from NB cell inoculation were significantly smaller than control tumors (n = 21) (CD31 *vs* control p= 0.047). B) Treatment with hCD31 mAb had no effect on survival of tumor bearing mice which was superimposable to that of control mice.

**Figure 2:**
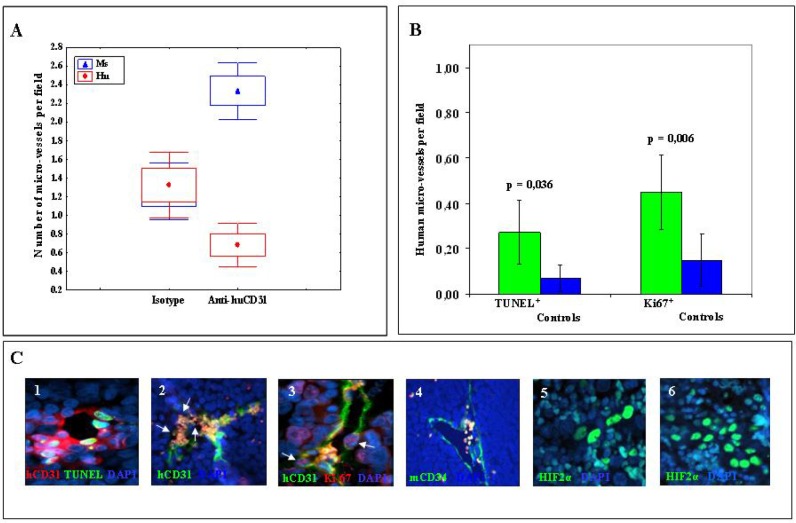
Endothelial micro-vessels in NB tumors from hCD31 mAb treated mice A) hCD31^+^ (red) and mCD34^+^ (blue) EM *per* field in tumors from hCD31 mAb *vs* isotype treated mice harvested after 18 days from tumor cell inoculation. Small circles represent median values, boxes represent 25%-75% and bars represent minimum and maximum values. B) Apoptotic (TUNEL^+^) and proliferating (Ki-67^+^) human EM increased significantly (p=0.036 and p=0.006, respectively) in tumors from hCD31 mAb treated *vs* control mice. Columns represent mean values; bars represent 95% Confidence Intervals. C) Immunofluorescence analysis of tumors from hCD31 treated mice: 1) double staining for hCD31 mAb (red) and TUNEL (green) detects human apoptotic EM, 2) focal micro-vascular destruction (white arrows) of hCD31^+^ EM (green); 3) double staining for hCD31 (green) mAb and Ki-67 (red) detects human proliferating EM (white arrows); 4) viable mCD34^+^ EM (green); 5, 6) NB tissue sections from control and hCD31 mAb treated mice stained with anti-hypoxia inducible factor-2α (HIF2α) mAb. NB tumors harvested after 18 days were studied (n = 7 tumors/group). Images in C illustrate representative data from one of at least three independent experiments.

Tumors from mice treated with hCD31 mAb (n=7) were significantly smaller than control tumors (n=7) (p= 0.047) (Fig. 1A), but treatment with hCD31 mAb did not prolong survival of tumor bearing (n=14) *vs* control (n=14) mice (Fig. 1B). Human EM density, assessed by hCD31 staining, decreased significantly (p= 0.011) in orthotopic tumors from hCD31 mAb treated (n=7) *vs* control (n=7) mice (Fig. 2A). Accordingly, apoptotic hCD31^+^ EM (defined as EM containing at least three TUNEL^+^ TDEC) increased significantly (p= 0.036) in the former (n=5) *vs* the latter (n=5) tumors (Fig. 2B and 2C, panel 1). Focal micro-vascular destruction and hemorrhagic areas were detected in tumors from hCD31 mAb treated mice (Fig. 2C, panel 2).

Double staining of NB tissue sections with hCD31 mAb and anti-Ki-67 mAb, that identifies proliferating cells, showed that hCD31^+^, Ki-67^+^ EM (defined as EM containing at least three Ki-67^+^ TDEC) increased significantly (p= 0.006) in tumors from hCD31 mAb treated vs control mice (Fig. 2B and 2C, panel 3), indicating the occurrence of human EM remodeling.

Mouse EM integrity was unaffected by hCD31 mAb treatment (Fig. 2C, panel 4) and mouse EM density was increased in tumors from hCD31 mAb treated *vs* control mice (p< 0.0001) (Fig. 2A).

### hCD31 mAb immunotherapy increases tumor hypoxia

TDEC depletion induced by hCD31 mAb treatment may reduce blood supply and increase tumor hypoxia. Hypoxia inducible factor-2α (HIF2α) is up-regulated in NB cells during hypoxic conditions and represents the most reliable hypoxic marker in NB [19]. In addition, HIF2α has been associated with developing endothelium [20].We stained by immunofluorescence orthotopic NB tissue sections from hCD31 treated (n=7) or control (n=7) mice with anti-HIF2α mAb [19]. The proportion of HIF2α^+^ cells was significantly higher in the former than in the latter tumors (61.9±3.22% *vs* 20.7± 6.18%, p= 0.0001) (Fig. 2C, panels 5 and 6).

In essence, TDEC contributed to NB vascularization since their targeting with hCD31 mAb reduced tumor size, decreased the number of human EM and increased hypoxia in tumor microenvironment.

### hCD31 mAb immunotherapy up-regulates expression of human pro-angiogenic genes in NB cells

**Figure 3:**
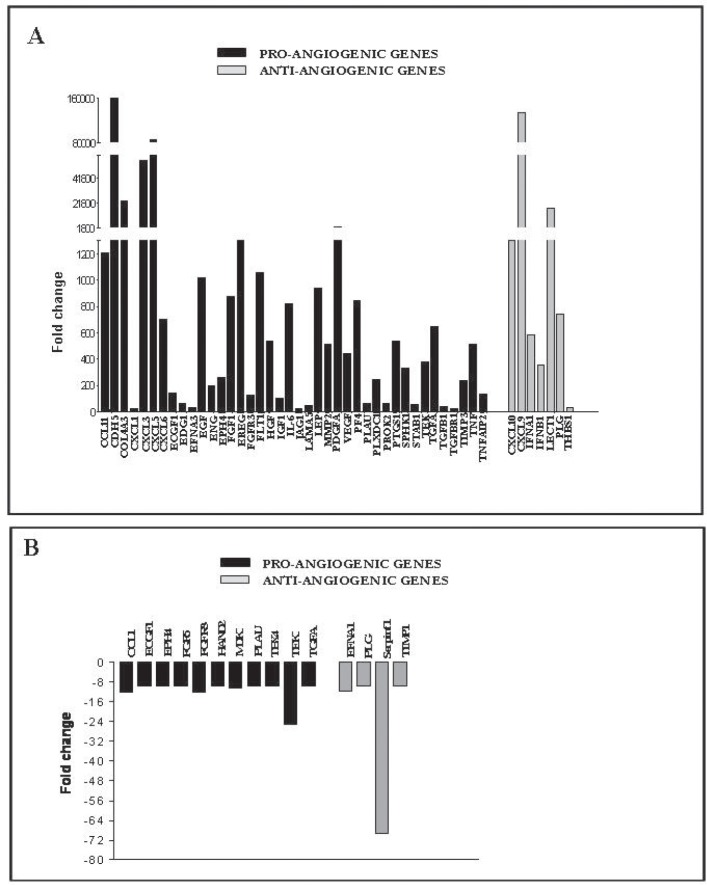
Human and mouse angiogenic gene expression in orthotopic NB tumors from anti-hCD31 mAb treated mice NB tumors from hCD31 mAb treated mice were harvested after 18 days. A) Human angiogenesis PCR array analysis of one representative NB tumor (out of four) from hCD31 mAb treated *vs* control mice. Pro-angiogenic genes, black bars; anti-angiogenic genes, grey bars. B) Mouse angiogenesis PCR array analysis of one representative NB tumor (out of four) from anti-hCD31mAb treated *vs* control mice. Pro-angiogenic genes, black bars; anti-angiogenic genes, grey bars. Assays for each experiment were performed in triplicate.

We next investigated the angiogenic phenotype of tumors from hCD31 mAb treated (n=4) *vs* control (n=4) mice using PCR arrays specific for selected human or mouse angiogenic transcripts. hCD31 mAb treatment caused up-regulation (range 1,000-160,000 fold) of the expression of different human pro-angiogenic genes including CCL11, CXCL3, CXCL5, cadherin 5 (CDH5), also known as vascular endothelial (VE)-cadherin, collagen type IVα3 (COL4A3), vascular endothelial growth factor (VEGF), platelet derived growth factor-A (PDGFA), fibroblast growth factor-1 (FGF1), TNF and IL-6 (Fig. 3A, black bars). High expression of these pro-angiogenic genes, the majority of which are not species-specific, likely accounts for the increased numbers of mouse-derived EM in NB tumors from h-CD31 mAb treated mice.

Two distinctive “signatures” related to VM [21] and epithelial mesenchymal transition (EMT) [22-24], respectively, were identified. In particular, VM-related transcripts included VE-cadherin (CDH5), collagen IV (COL4A), endothelial differentiation receptor (EDG1), IL-6, TNF-induced protein 2 (TNFAIP2), the matrix metalloproteinase MMP2, jagged-1 (JAG1), i.e. the ligand for the receptor Notch-1, and thrombospondin-1 (THBS1) [21]. EMT-related transcripts included epidermal growth factor (EGF), hepatocyte growth factor (HGF), insulin growth factor-1 (IGF-1), TNF, CXCL5, IL-6, FGF-1, PDGFA and MMP2 [22-25].

Expression of some human anti-angiogenic genes including interferon (IFN)A1, IFNB1, CXCL9, CXCL10 and Leukocyte cell derived chemotaxin-1 (LECT1) was also increased following hCD31 treatment (Fig. 3A, grey bars), perhaps in response to massive up-regulation of pro-angiogenic genes. In contrast, expression of mouse angiogenesis related genes was unaffected or dampened by hCD31 treatment (Fig. 3B).

**Figure 4:**
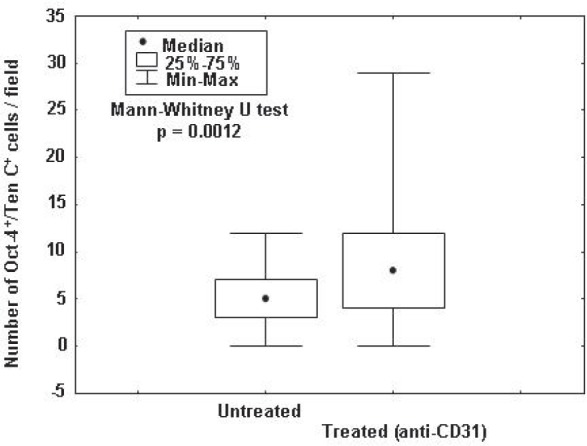
TNC^+^/Oct-4^+^ NB cell enumeration in tumors from hCD31 mAb treated mice NB tumors from hCD31 mAb treated (n = 5) or control (n = 5) mice were harvested after 18 days. Numbers of TNC^+^/Oct-4^+^ cells, that represent TDEC progenitors, *per* field were assessed by immunofluorescence. Small circles represent median values, boxes represent 25%-75% and bars represent minimum and maximum values.

We next asked whether human TDEC targeting altered the number and/or proliferative status of TNC^+^/Oct-4^+^ NB vascular progenitor cells [10], identified by TNC surface staining, and *MYCN* FISH. TNC^+^ NB cells carrying *MYCN* amplification, that showed a perivascular distribution [10], increased significantly in tumors from hCD31 treated (n=5) *vs* control (n=5) mice (p= 0.0012) (Fig. 4). Furthermore, the fraction of proliferating (Ki-67^+^) TNC^+^ cells was 8% (11/141) in hCD31 treated and 3% (5/154) in control mice (p< 0.0001; 95% Binomial Exact Confidence Interval).

Thus, TDEC remodeling triggered by hCD31 mAb-driven depletion of TDEC caused an increase of cycling TNC^+^ vascular progenitor NB cells, likely setting the stage for TDEC replenishment.

### NB vascular progenitor cells express the neural Tenascin C isoform A1, A2, A4, B

**Figure 5:**
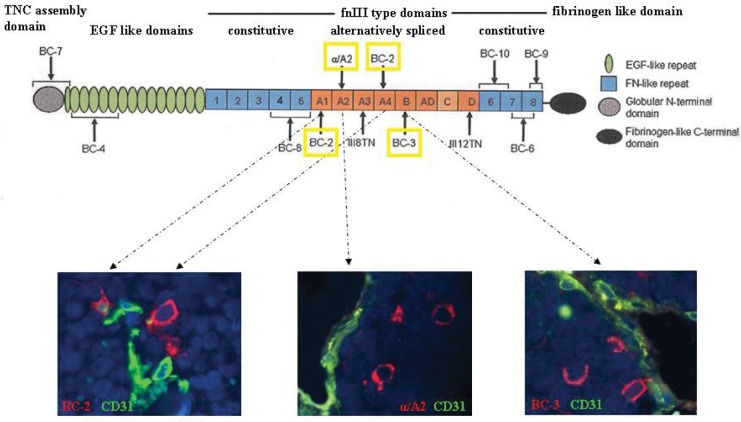
Expression of the large TNC isoform containing the A1, A2, A4, and B FnIII domains Upper panel shows schematic representation of the TNC protein. The domain structure of TNC comprising N-terminal assembly domain is followed by epidermal growth factor (EGF)-like repeats, the fibronectin type III (fnIII)-like repeats and the fibrinogen-like domain. The fnIII region consists of eight conserved repeats, designated 1 to 8, and up to nine alternatively spliced fnIII repeats designated as letters A to D. Lower panels show the immunofluorescence images after double staining of orthotopic tumor tissue sections formed by HTLA-230 cells in immunodeficient mice (n=5) with BC-2 (A1 and A4 specific), α/A2 (A2 specific) and BC-3 (B specific) mAbs (all in red), and hCD31 mAb (green). Representative images of one out of three experiments performed are shown.

TNC is encoded by a single gene and its expression is regulated by a single promoter [26]. Structurally and functionally different human TNC isoforms are generated by alternative splicing of the TNC transcript [26]. TNC is a multi-modular protein with a cysteine-rich assembly domain, epidermal growth factor (EGF)-like domains, several fibronectin type III (FnIII) domains, and a fibrinogen-like domain [25]. Up to six additional domains can be inserted between the fifth and sixth domain by alternative splicing domains (A1 to A4, B, C and D) [18, 27]. To identify what isoform(s) of TNC was expressed by NB progenitor cells, we used a series of murine mAbs specific for different alternatively spliced domains of this molecule [18]. Only the TNC isoform containing the alternatively spliced FnIII domains A1, A2, A4, and B was consistently detected in TNC^+^ NB vascular progenitor cells present in orthotopic NB tumors (n=4) (Fig. 5). This large TNC isoform is selectively expressed in neural stem/progenitor cells [28].

### hCD31 mAb immunotherapy induces epithelial-mesenchymal transition in orthotopic NB tumors

Detection of an EMT “signature” in NB cells from hCD31 mAb treated mice prompted experiments in which tumor tissue sections were stained with mAbs to Twist-1, the master regulator of EMT [29], N-cadherin and E-cadherin.

**Figure 6:**
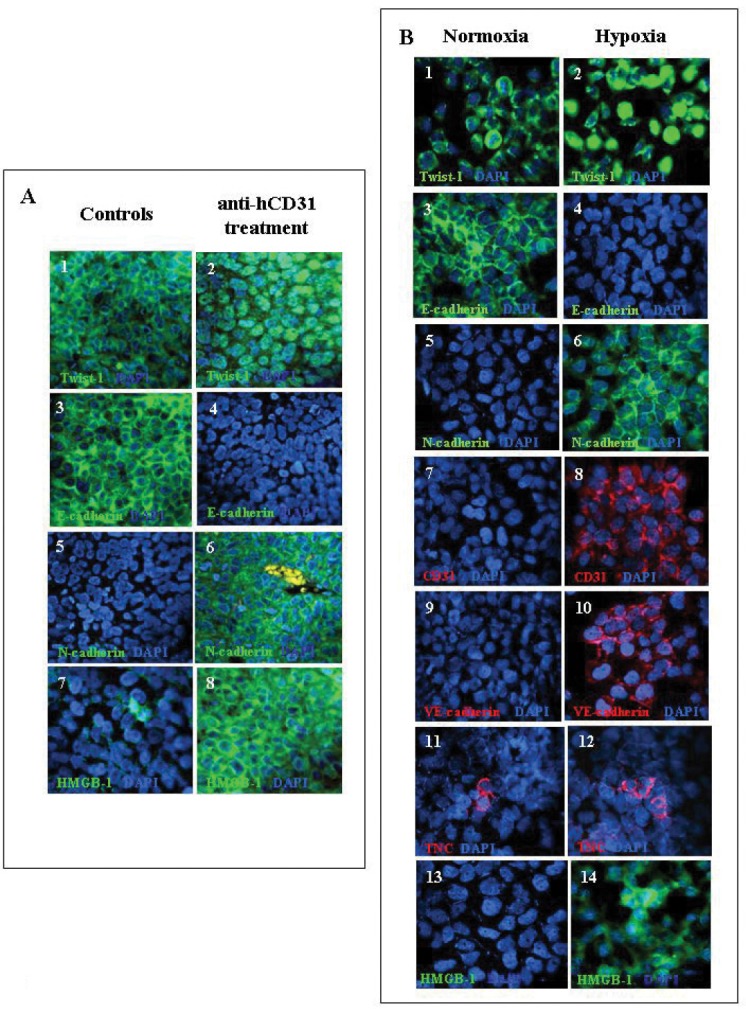
Expression of EMT proteins in orthotopic NB tumors from hCD31 mAb treated mice and in human NB cell lines incubated *in vitro* under normoxia or hypoxia A) Immunofluorescent staining of paraffin sections of orthotopic NB tumors from mice treated with hCD31 (n= 7) or control mice (n=7). Staining for Twist-1 (1, 2), E-cadherin (3, 4), N-cadherin (5, 6), HMGB-1 (7, 8). The Figure shows one representative experiment out of the four performed.. B) HTLA-230 cells were incubated in hypoxic or normoxic environment for 48h. Expression of EMT-related and endothelial markers, as well as of HMGB-1, was detected by immunofluorescent staining. Staining for Twist-1 (1, 2), E-cadherin (3, 4), N-cadherin (5, 6), hCD31 (7, 8), VE-Cadherin (9, 10), TNC (11, 12) and HMGB-1 (13, 14). Nuclei are stained with DAPI (blue). Original magnification 40x. One representative experiment out of the four performed is shown.

Most tumor cells from control mice (n=7) showed cytoplasmic expression of Twist-1 (83±5%), consistent with its transcriptional inactive state [29] (Fig. 6A, panel 1). The same cells expressed E-cadherin (98±2%) (Fig. 6A, panel 3) but not N-cadherin (Fig. 6A, panel 5).

In contrast, tumors from hCD31 mAb treated mice (n=7) showed in most of the cells nuclear expression of Twist-1 (79±7%), indicative of ongoing transcriptional activity [29] (Fig. 6A, panel 2). In addition, NB cells displayed expression of N-cadherin (98±3%) (Fig. 6A, panel 6) but not E-cadherin (Fig. 6A, panel 4). These results confirmed the onset of EMT [30] in tumors from hCD31 mAb treated mice.

Hypoxia may up-regulate the expression of high-mobility group box protein-1 (HMGB-1) [31], that acts as extracellular signaling molecule during inflammation, angiogenesis, cell differentiation, cell migration, and tumor metastasis [32-35]. Indeed, HMGB-1^+^ cells were significantly increased in tumors from hCD31 mAb treated (n=7) *vs* control (n=7) mice (77.3±8.2% *vs* 11.0±2.7%, p= 0.0004). HMGB-1 was always detected in the cytoplasm of NB cells (Fig. 6A, panels 7 and 8), suggesting a role of this molecule in the stimulation of cell motility [32-35].

### *In vitro* culture of HTLA-230 NB cells under hypoxic conditions induces expression of epithelial mesenchymal transition-related and endothelial cell-related markers, as well as of HMGB-1

In order to investigate the *in vitro* conditions allowing expression of EMT-related and endothelial markers in NB cells, HTLA-230 cells were incubated in hypoxia (1% O_2_) or normoxia for 24-72 h. In four different experiments, cytoplasmic Twist-1 was detected in 80±13% cells under normoxia only (control) (Fig. 6B, panel 1), whereas nuclear Twist-1 was detected in 76±5% HTLA-230 cells under hypoxia only (Fig. 6B, panel 2). Hypoxia induced down-regulation of E-cadherin (<1%), while strong basal expression of the latter marker (mean± SD 82±7%) was detected in normoxic cells (Fig. 6B, panels 3 and 4, respectively). Conversely, hypoxia induced *de novo* expression of N-cadherin (mean ±SD 85±10%) that was detected in less than 1% normoxic cells (Fig. 6B, panels 5 and 6). Under hypoxia, HTLA-230 cells acquired expression of the endothelial-specific markers CD31 (mean ±SD 35±9% ) (Fig. 6B, panels 7 and 8) and VE-cadherin (mean ±SD 72±6%) (Fig. 6B, panels 9 and 10), that were undetectable in normoxic cells. Furthermore, TNC^+^ cells, that serve as TDEC progenitors [10], were significantly increased in hypoxic compared to normoxic NB cells (means ±SD: 20.0±4.3% *vs* 10.0±2.7%, p= 0.006) (Fig. 6B, panels 11 and 12). Finally, most HTLA-230 cells cultured in hypoxia acquired the expression of HMGB-1, that was virtually undetectable in normoxic cells.

These data establish definitively that hypoxia induces expression of EMT-related and endothelial markers in NB cells.

### HMGB-1 mimics hypoxia *in vitro*

**Figure 7:**
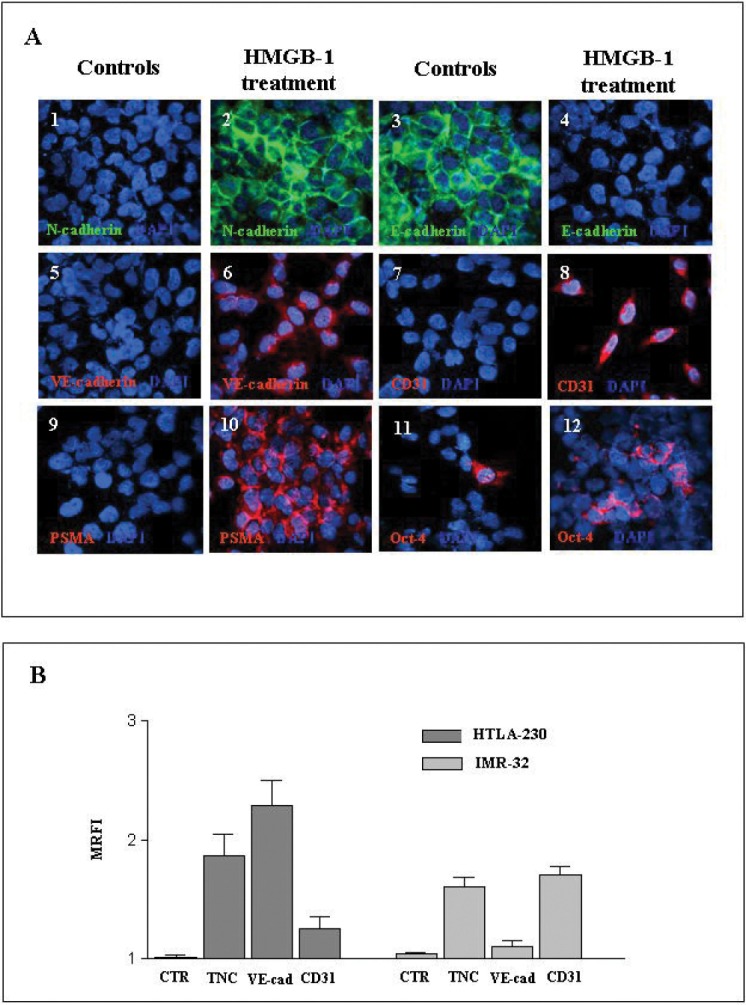
Incubation of NB cell lines *in vitro* with HMGB-1 induces expression of EMT-related and endothelial markers A) Immunofluorescence staining of cytospins of the HTLA-230 cell line cultured for 48 h in the absence or presence of human recombinant HMGB-1 protein (10μg/ml). Cytospins were stained for N-cadherin (1, 2), E-cadherin (3, 4), VE-cadherin (5, 6), CD31 (7, 8), PSMA (9, 10) and Oct-4 (11, 12). Nuclei are stained with DAPI (blue). Original magnification 40x. One representative experiment out of the four performed is shown..B) Expression of TNC, VE-cadherin and CD31 were evaluated by flow cytometry in HTLA-230 and IMR-32 cell lines cultured in the presence or absence of HMGB-1 as above. Results are expressed as MRFI and represent means ±SD from three independent experiments.

Since HMGB-1 induces EMT in lung [32] and renal [34] fibrosis, we finally investigated whether HMGB-1 behaved similarly in our human NB models under normoxic conditions. In four different experiments, HTLA-230 cells were cultured with HMGB-1 for 24-72 h. Fig. 7A shows that HTLA-230 cells cultured with HMGB-1 acquired the expression of N-cadherin and lost that of E-cadherin, indicative of an ongoing EMT. The same cells expressed *de novo* VE-cadherin (mean ±SD 87±4.3%), CD31 (mean ±SD 30±2.5%), and PSMA [10] (mean ±SD 75±1%), witnessing the occurrence of trans-endothelial differentiation of NB cells. Accordingly, TDEC progenitors expressing Oct-4 both in the nucleus and the cytoplasm were doubled in HTLA-230 cells incubated with HMGB-1 compared to control (means ±SD: 18.0±3% *vs* 9.0±3.2%, p= 0.008) (Fig. 7A).

When HTLA-230 and IMR-32 NB cell lines were treated with HMGB-1 and analyzed by flow cytometry (n=3 experiments), both cell lines variably up-regulated the expression of TNC, VE-cadherin and CD31, thus confirming that HMGB-1 induced the acquisition of EMT-related (TNC) and endothelial (VE-cadherin and CD31) markers (Fig. 7B).

## DISCUSSION

Primary NB tumors may contain a variable number (range 20-78%) of TDEC carrying the same genomic alteration as tumor cells [10]. TNC^+^ perivascular NB cells serving as TDEC progenitors have been identified in primary NB samples, metastatic bone marrow aspirates, NB cell lines, and orthotopic tumors formed by these cell lines in immunodeficient mice [8-10].

Studies aimed at targeting TDEC, that are genetically unstable and contribute to tumor progression [11], have shown that thalidomide, sirolimus, curcumin, isoxanthohumol or resveratrol inhibited TDEC formation in various pre-clinical models [36]. Differentiation of CD133^+^ glioblastoma stem cells into TDEC [37-39] was unaffected by anti-VEGF mAb, but blocked by γ-secretase inhibition or Notch-1 silencing [39].

Here we have targeted for the first time human TDEC *in vivo* with the cytotoxic hCD31 mAb Moon-1, taking advantage of its high specificity witnessed by the lack of any reactivity with host-derived EC. Significant decrease of TDEC due to apoptosis and reduction of tumor size unambiguously proved that TDEC contributed to NB growth. However, tumors harvested at the end of immunotherapy cycles displayed active vascular remodeling, as shown by detection of proliferating TDEC. In this respect, treatment with VEGF inhibitors or VEGF gene inactivation in preclinical mouse models of cancer were previously found to increase tumor invasiveness and metastasis, as well as EC proliferation, that could play a role in a putative rebound dampening anti-angiogenic treatment efficacy [40-42].

Hypoxia-inducible factor renders cells capable of surviving in hypoxia and stimulates endothelial cell growth [43]. On one hand, hypoxia inhibits the mTOR pathway, mTOR-dependent translation of HIF-1 and prevents senescent phenotype [44]. On the other hand, hypoxia strongly posttranslationally induces HIF-1, which increases secretion of mitogens and cytokines, markers of cellular senescence and promotes it [43]. We reasoned that hypoxia could drive vascular remodeling in NB tumors from mice treated with hCD31 mAb [45, 46]. Indeed, a highly significant increase of HIF-2α expression was detected in tumors from hCD31 mAb treated mice, indicating that TDEC targeting resulted into generation of a strongly hypoxic tumor microenvironment. Hypoxia was previously shown to induce expression of VM-related genes [21]. Indeed, up-regulation of the expression of VM-related and other pro-angiogenic genes was detected by PCR array in tumors from hCD31 mAb treated mice. Angiogenic factors, which are produced by tumor cells in response to hypoxia, reduce the pro-apoptotic potency of chemotherapy on endothelial cell [47]. By activating hypoxic response in tumor cells, antiangiogenic therapy may promote metastasis and invasion [47].

Hypoxia is also an inducer of EMT, characterized by loss of cell junction and gain of migratory behavior [30, 33]. *Twist-1* is a basic helix-loop-helix transcription-factor that promotes EMT [48-50] through i) repression of E-cadherin [48], leading to disassembly of adherens junctions and increased migratory potential [50], and ii) up-regulation of mesenchymal markers, as N-cadherin and TNC [49]. In our experiments, tumors from hCD31 mAb treated mice showed nuclear translocation of Twist-1, lost E-cadherin expression and acquired N-cadherin expression, indicating the occurrence of hCD31 mAb-initiated EMT. These findings were corroborated by the results of PCR array analysis of tumors from hCD31 mAb *vs* isotype treated mice showing the presence of an EMT-related “signature” in the former tumors.

Damage associated molecular patterns (DAMPs) are released by dying cells [51]. The main DAMP is HMGB-1 [52, 53], that promotes angiogenesis, evasion of programmed cell death, self-sufficiency in growth signals, insensitivity to inhibitors of growth, autophagy, and tissue invasion and metastasis [52-54]. Tumor angiogenesis is enforced by autocrine regulation of HMGB-1 in EC through increased expression of key pro-angiogenic genes as PDGF-A, FGF and MMP-2 [55], that we found to be strongly up-regulated in tumors from hCD31 mAb treated mice. We asked whether the latter tumors expressed HMGB-1. Indeed, most tumor cells from hCD31 mAb treated mice expressed HMGB-1, that was barely detectable in control tumors, suggesting that hypoxia augmented by hCD31 mAb treatment up-regulated HMGB-1 [52-54].

The results of *in vivo* experiments were confirmed by *in vitro* studies with NB cell lines incubated in hypoxia. These cells i) acquired expression of N-cadherin and nuclear Twist-1 and lost that of E-cadherin, ii) expressed *de novo* the EC-related markers CD31 and VE-cadherin, as well as HMGB-1, and iii) up-regulated the expression of TNC that, beside representing an EMT marker [56], identifies NB TDEC progenitors [10]. The latter finding is consistent with the significant increase of TNC^+^/Oct-4^+^ cycling TDEC progenitors observed in tumors from hCD31 mAb treated mice.

HMGB-1 can induce EMT in human kidney tubular epithelial cells [34] and mouse alveolar epithelial cells [32]. We found that HMGB-1 induced expression of EMT and EC markers in NB cells cultured in normoxia, thus mimicking the effects of hypoxia. HMGB-1 conceivably bound to NB cells through Toll-like receptor 4 (TLR-4), and/or RAGE [57], that have been shown in previous studies to be expressed by these cells [58]. It is tempting to speculate that HMGB-1 synergizes with hypoxia sub-serving the same or similar functions in less hypoxic or normoxic areas of the tumor mass.

Taken together, our results indicate that hypoxia-driven enhancement of VM and onset of EMT represent adaptive mechanisms to the perturbation of tumor microenvironment caused by hCD31 mAb treatment. Notably, in this respect, nuclear translocation of Twist-1 [29] initiated VM in hepatocellular [29, 50] and colorectal [59] carcinomas, showing that EMT and VM are linked.

Additional mechanisms beside hypoxia may contribute to the onset of EMT in tumors from hCD31 mAb treated mice. IL-6, whose expression was up-regulated in the latter tumors, is a well known inducer of EMT and mediates trastuzumab resistance in breast cancer [60]. Anaphylotoxins as C3a and C5a, generated in our study by hCD31 mAb-mediated complement activation, regulate EMT in non-malignant disease models [61, 62].

**Figure 8:**
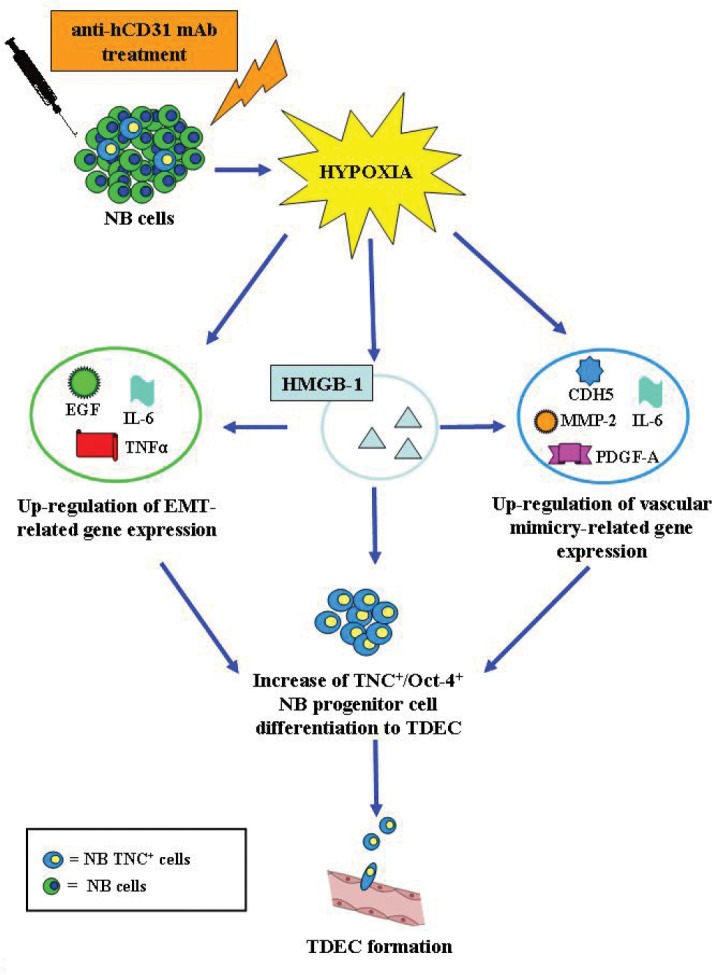
A model for hypoxia-driven EMT and trans-endothelial differentiation of NB cells TDEC targeting with cytotoxic hCD31 mAb enhances tumor hypoxia, which in turn induces EMT and endothelial trans-differentiation of tumor cells. Both of the latter phenomena increase the proportion of TNC^+^/Oct-4^+^ NB cells, that differentiate into TDEC responsible for the vascular remodeling observed in tumors from hCD31 treated mice. In addition, hCD31 mAb-driven hypoxia promotes in NB cells expression of HGMB-1 that induces by itself EMT and endothelial trans-differentiation of tumor cells, thus mimicking the effects of hypoxia and serving as an amplification loop. All of these mechanisms in combination account for the refractoriness of NB tumors to TDEC targeting with hCD31 mAb.

In conclusion, we propose the following model (Fig. 8). TDEC targeting with cytotoxic hCD31 mAb enhances tumor hypoxia, which in turn induces EMT and promotes endothelial trans-differentiation of tumor cells. Both of the latter phenomena increase the proportion of TNC^+^/Oct-4^+^ NB cells, that differentiate into TDEC responsible for the vascular remodeling observed in tumors from hCD31 mAb treated mice. In addition, hCD31 mAb-driven hypoxia promotes in NB cells expression of HGMB-1 that induces by itself EMT and endothelial trans-differentiation of tumor cells, thus serving as an amplification loop of hypoxia (Fig. 8). Altogether these mechanisms account for the refractoriness of NB tumors to TDEC targeting with hCD31 mAb. A potential strategy to exploit hypoxia for therapeutic purposes might be to combine an antiangiogenic treatment with inactive pro-drugs that are activated by hypoxia [47].

## METHODS

### Orthotopic mouse model of human neuroblastoma

Seven/eight week old female athymic nude (*nude/nude*) mice from Harlan Laboratories (Harlan Italy, S. Pietro al Natisone, Italy) were housed in sterile enclosures under specific pathogen-free conditions. Mice were subjected to laparotomy, and injected with HTLA-230 cells (1.5×10^6^ cells in 20 μl of saline solution) in the left adrenal gland [10]. Mice were anesthetized with ketamine (Imalgene 1000, Merial Italia SpA., Milan, Italy) and sacrificed by cervical dislocation. All procedures involving animals were performed in the respect of the National and International current regulations (D.lvo 27/01/1992, n° 116, European Economic Community Council Directive 86/609, OJL 358, Dec. 1, 1987) and were reviewed and approved by the licensing and Ethical Committee of the IRCCS-AOU San Martino-IST National Cancer Research Institute, Genoa, Italy, and by the Italian Ministry of Health.

### Treatment of neuroblastoma bearing mice with hCD31 mAb

Tumor bearing mice were allocated randomly into three groups of seven mice each to receive intravenously (iv) the hCD31 Moon-1 purified mAb (IgG_2_, 450 μg/mouse) [17], or isotype-matched IgG_2_ irrelevant mAb (450 μg/mouse) for two weeks starting from day 2 after tumor cell inoculation. Treatment was administered every three days for a total of five injections. This protocol was repeated in three different experiments.

### Quantification of tumor growth

Mice were sacrificed i) when they showed signs of poor health to evaluate survival or ii) the day after the conclusion of last cycle of antibody treatment, i.e. day 18, in order to measure tumor volume and perform histological and immunohistochemical studies. Tumor volume was measured with a caliper using the following formula: Volume = π/6 × (w1 × w2) (w1= largest tumor diameter; w2 smallest tumor diameter). For histological analyses tissue sections were stained with hematoxylin and eosin.

### Immunofluorescence analysis and quantifications of micro-vessel density

Indirect immunofluorescence was performed on formalin-fixed, paraffin embedded or on cryopreserved tissue sections from mice sacrificed, or on cytospins of the HTLA-230 cell line as previously described [10]. Paraffin sections (2-4 μm thick) were processed by standard deparaffinization with xylene and hydrated in a descending ethanol series to double-distilled water. Antigen retrieval on formalin-fixed tissue section was performed using Sodium-Citrate buffer (pH 6.0). Indirect immunofluorescence was performed using the following mouse monoclonal antibodies (mAbs): anti-human(h)CD31 (diluted 1/50; Dako Cytomation, Hamburg, Germany), anti-mouse(m)CD34 (1/50; Novus Biologicals, Littleton, CO, USA), anti-hOct-4 (1/50, Clone 3A5; Abcam Inc, Cambridge, USA), anti-hTenascin C (1/50; Millipore, Milan, Italy), anti-Ki-67 (1/60; Dako Cytomation), anti hE-cadherin (1/25, Clone EP700Y; Millipore), anti hN-cadherin (1/25, Clone EPR1792Y; Millipore), anti-hTwist-1 (1/50, Sigma-Aldrich, St. Louis, MO, USA), anti hHMGB-1 (1/20, Clone 1B11, Abnova, Taipei, Taiwan), anti-hVE-cadherin (1:50; Chemicon International, Millipore, Billerica, MA, USA), and anti-HIF2α (1:100; Novus Biologicals) were used. In addition, we used the following mouse mAbs (1/20 dilution) specific for the different epitopes of hTNC, generously donated by Dr L. Zardi, Syrius Biotech, Genoa, Italy: anti BC-7, anti BC-4, anti BC-8, anti BC-6, anti BC-9, anti BC-2, anti-III8TN, anti-α/A2, anti BC-3, anti-III12TN [18]. Isotype-matched nonbinding mAbs were used in all antibody-staining experiments to exclude non specific reactivities. Slides were incubated with primary antibodies overnight at 4° C. Secondary antibodies were goat anti-mouse IgG conjugated to Alexa-488 or goat anti-rabbit IgG Alexa-568 (1:200; Invitrogen, Germany). After washing, the slides were counterstained with 4′,6′-diamidino-2- phenylindole (DAPI, Sigma-Aldrich, Milan, Italy) and cover-slipped.

EM density was assessed by anti-hCD31 or anti-mCD34 staining and examination of twenty to fifty microscopic fields (0.5 mm^2^) per tumor. We have shown in a previous study that the anti-hCD31 or anti-mCD34 mAbs used in this study are rigorously species-specific [10]. The most intense vascular areas (hotspots) were selected subjectively from each tumor section. EM with a clearly defined lumen or well-defined linear vessel shape were taken into account for EM counting.

### Detection of apoptotic or proliferating endothelial cells

Apoptosis was evaluated using *in situ* Cell Death Detection Kit (Roche Diagnostics, Penzberg, Germany). The Terminal Deoxynucleotidyl Transferase-Mediated dUTP Nick-End Labeling (TUNEL) reaction was carried out by incubating tumor tissues with FITC-dUTP labeled nucleotides and terminal deoxynucleotidyl transferase (TdT), which catalyzes polymerization of labeled nucleotides to free 3′-OH DNA ends. Apoptotic or proliferating cells were detected by staining for TUNEL or Ki-67, respectively, in combination with hCD31. Apoptotic or proliferating EM were operationally defined as containing at least three TUNEL^+^ or Ki-67^+^ cells, respectively.

### Fluorescent In Situ Hybridization (FISH)

Interphase FISH was performed on paraffin-embedded tumor tissue sections as described [10] using rhodamine-labeled *MYCN*-specific DNA probe (QBIOgene Inc, Hamburg, Germany) and mouse Cot-1 DNA probe (Life Technologies Gibco BRL, Paisley, Scotland).

### PCR-Array profiling

RNA was extracted using TRIZOL® from Invitrogen (Carlsbad, CA, USA) and reverse transcribed by the ReactionReady™ First Strand cDNA Synthesis kit (SuperArray Bioscience Corporation). Human and murine angiogenesis RT^2^ Profiler™ PCR Array and RT^2^ Real-Timer™ SyBR Green/ROX PCR was performed according to the instructions of the manufacturer on ABI Prism™ 7700 Sequence Detector (Applied Biosystems).

### Cell culture and human recombinant HMGB-1 protein treatment of HTLA-230 and IMR-32 cell lines

HTLA-230 and IMR-32 cells were maintained as previously described [10]. HTLA-230 and IMR-32 NB cells were cultured on glass slide containing dishes (Millipore) with or without human recombinant HMGB-1 protein (10μg/ml, Sigma-Aldrich) for 72 h. E-cadherin, N-cadherin, Twist-1, TNC, CD31 and VE-cadherin expression were assessed by immunofluorescence staining.

### Flow cytometry

TNC, CD31 and VE-cadherin expression was investigated on HTLA-230 and IMR-32 cell lines cultured with or without HMGB-1 (10μg/ml) by flow cytometry. Cells were first incubated for 30 min at 4° C with primary mAbs, then for 20 min with a polyclonal rabbit anti-mouse FITC-conjugated mAb (Dako Cytomation, Denmark). An isotype-matched primary mAb (Merk Millipore, Billerica, MA) was used as negative control. Samples were analyzed by Gallios flow cytometer and Kaluza software (Beckman Coulter, Milano, Italy). Results are expressed as MRFI calculated as ratio between MFI of specific mAb and MFI of irrelevant isotype-matched mAb.

### *In vitro* hypoxia models

HTLA-230 and IMR-32 cell lines were incubated for 24, 48 and 72 h at 37° C. in a hypoxia incubator (Invivo2 Hypoxia Workstation, Ruskinn Technology, UK) which was flushed by 1% O_2_.

### Image Analysis

Digital images were collected using a Nikon E-1000 fluorescence microscope (Nikon Instruments, Tokyo, Japan) equipped with appropriate filter sets and the Genikon imaging system software (Nikon Instruments).

### Statistical analysis

Statistical significance of differences between experimental and control groups was determined by ANOVA with Tukey's multiple comparison test using GraphPad Prism 3.0 software (GraphPad Software, Inc.). Survival curves were constructed by using the Kaplan-Meier method. Survival in different treatment groups was compared by using Peto's log-rank test in StatsDirect 0.1 statistical software (CamCode). Tumor volumes were compared by non parametric Mann Whitney test, one tailed (Confidence intervals 90%). P< 0.05 was considered statistically significant.

### Funding

This study was supported by Project Ricerca Finalizzata, Ministero della Salute “The primary and metastatic cancer stem cells microenvironment in neuroectodermal tumors: studies in human neuroblastoma and melanoma” (grant number G21J11000040001), Project Regione Liguria “Endotelio di derivazione tumorale: caratterizzazione e targeting immunologico a fini terapuetici” (grant number G31J10000970009), “5 per mille IRPEF Finanziamento della Ricerca Sanitaria”, and Finanziamento Ricerca Corrente. CC is the recipient of a fellowship from Umberto Veronesi Foundation, Milano, Italy.
