# Supplementary material for: Failure of anti tumor-derived endothelial cell immunotherapy depends on augmentation of tumor hypoxia
Source: Oncotarget. 2014 May 26;5(21):10368–81. doi: 10.18632/oncotarget.2015 (PMC4279379; doi:10.18632/oncotarget.2015)
Supplement: Supplementary file 1 [file oncotarget-05-10368-s001.pdf]

Failure of anti tumor-derived endothelial cell immunotherapy depends on augmentation of tumor hypoxia – Pezzolo et al

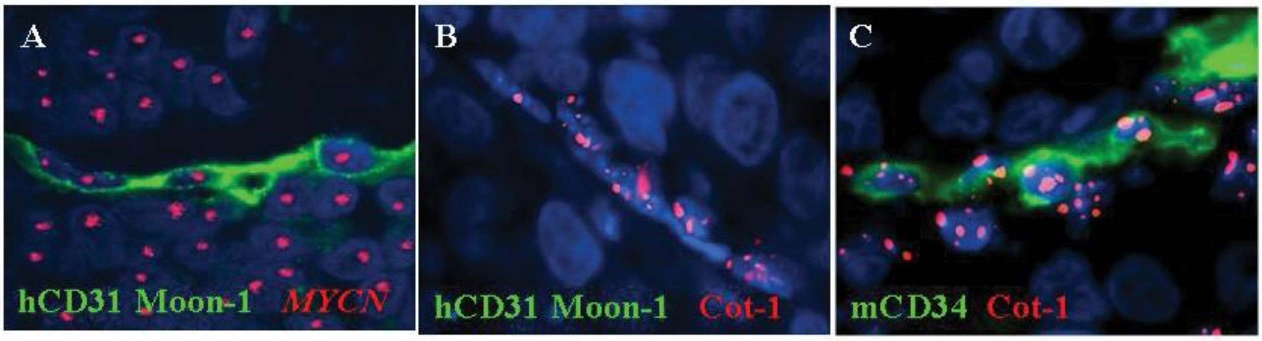

Figure S1: Anti-hCD31 Moon-1 mAb detects an epitope expressed only on human endothelial cells

A) An endothelial micro-vessel lined by human CD31$^+$ cells (green) carrying *MYCN* amplification (red). **B)** A mouse endothelial micro-vessel detected by Cot-1 FISH is negative for human CD31 staining. **C)** An endothelial micro-vessel lined by murine CD34$^+$ cells (green) showing red hybridization signals with mouse Cot-1 DNA probe. Nuclei are stained with DAPI (blue). Original magnification 100x.
